# Insight into Central Asian flora from the Cenozoic Tianshan montane origin and radiation of *Lagochilus* (Lamiaceae)

**DOI:** 10.1371/journal.pone.0178389

**Published:** 2017-09-20

**Authors:** Ming-Li Zhang, Xiao-Qing Zeng, Stewart C. Sanderson, Vyacheslav V. Byalt, Alexander P. Sukhorukov

**Affiliations:** 1 Key Laboratory of Biogeography and Bioresource in Arid Land, Xinjiang Institute of Ecology and Geography, Chinese Academy of Sciences, Urumqi, China; 2 Institute of Botany, Chinese Academy of Sciences, Beijing, China; 3 College of Life Sciences, Central China Normal University, Wuhan, China; 4 Shrub Sciences Laboratory, Rocky Mountain Research Station, Forest Service, U.S. Department of Agriculture, Provo, Utah, United States of America; 5 Komarov Botanical Institute, Russian Academy of Sciences, St. Petersburg, Russia; 6 Biological Faculty, Moscow Lomonosov State University, Moscow, Russia; Institute of Botany, CHINA

## Abstract

The Tianshan Mountains play a significant role in the Central Asian flora and vegetation. *Lagochilus* has a distribution concentration in Tianshan Mountains and Central Asia. To investigate generic spatiotemporal evolution, we sampled most *Lagochilus* species and sequenced six cpDNA locations (*rps*16, *psb*A-*trn*H, *mat*K, *trn*L-*trn*F, *psb*B-*psb*H, *psb*K-*psb*I). We employed BEAST Bayesian inference for dating, and S-DIVA, DEC, and BBM for ancestral area/biome reconstruction. Our results clearly show that the Tianshan Mountains, especially the western Ili-Kirghizia Tianshan, as well as Sunggar and Kaschgar, was the ancestral area. Ancestral biome was mainly in the montane steppe zone of valley and slope at altitudes of 1700–2700 m, and the montane desert zone of foothill and front-hill at 1000–1700 m. Here two sections *Inermes* and *Lagochilus* of the genus displayed “uphill” and “downhill” speciation process during middle and later Miocene. The origin and diversification of the genus were explained as coupled with the rapid uplift of the Tianshan Mountains starting in late Oligocene and early Miocene ca. 23.66~19.33 Ma, as well as with uplift of the Qinghai-Tibetan Plateau (QTP) and Central Asian aridification.

## Introduction

*Lagochilus* belongs to Lamiaceae, the mint Family. The chemical structure and components of many *Lagochilus* species were surveyed due to their medicinal uses [1–3], and some species were put into the Red Data Book of the former USSR because of their rarity [4]. The chromosome number in *Lagochilus* was reported as 2n = 22, but only in one species, *L*. *schugnanicus* Knorring [5]. Within the family, *Lagochilus* belongs to subfamily Lamioideae [6] and tribe Leonureae Dumort. Although recent molecular phylogenies [7–9] shew that generic circumscription of Lamioideae is variable, the placement of the tribe Leonureae is always fixed. Within tribe Leonureae, *Lagochilus* is the largest genus with ca.44 species [10–12]. This genus has the distinct taxonomical traits, with spiny bracteoles longer than calyces, spines usually present in the leaf axils, and a densely villous, 2-lobed, long and narrow posterior corolla lip [6]. *Lagochilus* is generally divided into two sections [10,11], firstly established by Fisher and Meyer in 1841 [12], section *Lagochilus* Fisch. et Mey. with spinous bracteoles at the leaf axils of sterile branches, and Sect. *Inermes* Fisch. et Mey., without such bracteoles. Taxonomic series ranks under two sections were only established in Flora USSR [10,12]. Ikramov investigated the classification, distribution, and community ecology of thirty-two species in Central Asia of the Former USSR [3], twenty neo-endemic species in Central Asia, where was inferred as region of origin. Extracted five species from *Lagochilus*, Knorring established a genus *Lagochilopsis* [13]. Liu revised Chinese classification of twelve species included mainly in Xinjiang[14]. Zuckerwanik rearranged the generic classification system [15], mainly added southern range species in Pakistan and Afghanistan around Hindukush. Jamzad treated Iranian species [16]. All classifications were fundamentally followed the framework of Knorring’ system [12]. However, there is no a comprehensive classification covering all species of *Lagochilus* so far.

Many molecular phylogenetic investigations of Lamiaceae paid attention to subfamilial or tribal levels [7–9,17,18], only a few concerned genera of Leonureae, and 1–3 species sampled from *Lagochilus*. Biogeographically, Roy and Lindqvist (2015) suggested an Oligocene-Miocene origin of the Lamioideae, with a crown age of ca. 23.9 Ma [8]. Tribe Leonureae was dated 9.4 Ma (cpDNA time tree) and 5.7 Ma (PPR time tree), and tribe Paraphlomideae at ca. 6.1 Ma (cpDNA time tree). Asia and the Mediterranean region were presumed as the centers of diversity and place of origin for many lamioid tribes.

Central Asia possess most *Lagochilus* species, mainly in arid and semiarid regions of China, Kazakhstan, Uzbekistan, Mongolia, Iran, Pakistan, Afghanistan, etc., and endemic to the Asian mountains, including the Tianshan and Pamir-Alai, as well as the surrounding Hindukush, Altai, and Caucasus ranges, etc. These mountains generally are the hotspots of global biodiversity [19]. In China, along the Tianshan Mountains, mainly the Ili Valley and the Karakoram Mountains, we can find *L*. *grandiflorus*, *L*. *platyacanthus* and *L*. *kaschgaricus*; and in the Altai Mountains, there are *L*. *diacanthophyllus*, *L*. *hirtus*, *L*. *bungei*, and *L*. *macrodontus*. A few, such as *L*. *ilicifolius*, extend to desert (sandy land) and steppe in northwestern China [10,20]. Thus, the montane distribution pattern of *Lagochilus* possibly provides a biogeographical case of origination from the Tianshan Mountains to the Central Asian flora, like other montane plant lineages [21], for example, *Atraphaxis* (Tianshan Mountains) [22], *Myricaria* (western Himalayas) [23], and *Calophaca* (Pamir) [24].

Since only 1–3 species sampled from *Lagochilus* in the previous phylogenies [7–9], and the phylogeography concerned ten species in China [20], clearly it is insufficient to confirm generic monophyly and morphological classification. Also, a dated age of tribe Leonureae with 9.4–5.7 Ma [8] seems rather young, and we want to know the exact diversification age and spatiotemporal evolution of *Lagochilus*. Therefore, using *Lagochilus* as a case of montane plant lineages, densely sampling species, and sequencing six cpDNA markers, we attempt to revisit Central Asian flora in this paper: to reconstruct phylogenetic relationships and ascertain major clades in *Lagochilus*; to explore its spatiotemporal diversification, and to discuss the biogeographical significance of the genus for the Central Asian flora. In addition, discussing evolutionary history of *Lagochilus* in the Tianshan Mountains and Central Asia, the palaeogeology and palaeoclimate events, such as Tianshan uplift, and Central Asian aridification etc. in the Cenozoic in these regions, should be linked so that explain the evolutionary dynamics.

## Material and methods

### Taxon sampling

We sampled and sequenced species of *Lagochilus*, *Panzerina*, and *Leonurus* of tribe Leonureae, see S1 Table. According to the recent phylogenetic scheme of Lamiaceae subfamily Lamioideae Harley [7], suitable outgroups for *Lagochilus* would extend to *Lamium* of tribe Lamieae, *Stachys* of tribe Stachydeae, *Physostegia* of tribe Synandreae, *Achyrospermum* of tribe Pogostemoneae, *Thymus*, *Origanum*, *Nepeta*, *Salvia*, *Hypenia*, *Lavandula*, and *Elsholtzia* of Lamiaceae subfamily Nepetoideae Kosteletzky, *Hemigenia* and *Prostanthera* of Lamiaceae subfamily Prostantheroideae Luersson, as well as *Acanthus* of family Acanthaceae and *Olea* of family Oleaceae. See S2 Table. Species samples and vouchers are deposited in five herbaria, see S1 Table.

We state that all use of species materials were permitted by the authority of these herbaria. Our study did not concern Human Subject Research or Animal Research. We can declare that the leaf materials did not come from conservation parks, and none of the samples involved endangered or protected species.

### DNA sequencing

Total genomic DNA was extracted using the CTAB method [25]. Six cpDNA sequences and selected primers were *rps16* [26]; *psb*A-*trn*H [27]; *mat*K [28]; *trn*L-trnF [29]; *psb*B-*psb*H [22]; *psb*K-*psb*I [30]. The polymerase chain reaction (PCR) was used for amplification of double stranded DNA. The 25 μl reaction system contained 0.25 μl of Ex Taq, 2.5 μl of 10× Ex Taq buffer (Mg2+ concentration of 25 mM), 2.0 μl of dNTP mix (2.5 mM concentration for each dNTP), 1 μl of the forward and reverse primers at 5 umol /μl, and 0.5 μl of template DNA. The protocol for amplification consisted of an initial hot start at 95°C for 2 min, followed by 30 cycles of denaturation at 94°C for 30 s, annealing at 52°C for 30 s, extension at 72°C for 90 s, and a final extension at 72°C for 10 min. PCR products were purified using the PEG precipitation procedure [31] and sequenced using an ABI PRISM 3770 Genetic Analyzer (Shanghai Shenggong Biological Engineering Technology & Service, Shanghai, China).

Sequences were aligned with CLUSTAL X software [32] and then adjusted manually. All gaps were treated as missing data. Finally, the combined 6-gene region data set comprised 4633 aligned nucleotide characters.

### Phylogenetic analysis and divergence time estimates

Congruence among the six cpDNA sequences was assessed by comparing topologies and support values from MrBayes analysis, employing Markov chain Monte Carlo runs of 20 million generations sampled every 1,000 generations. Majority rule (>50%) consensus trees were constructed after removing the burn-in samples (the first 10% of sampled trees). The Bayesian tree showed no major incongruence supported by posterior probabilities of 0.95 or greater, therefore, we combined the six sequence datasets for further phylogenetic examination.

Phylogenetic analyses were performed using Maximum Likelihood (ML) and Bayesian inference. ML analysis was performed with PAUP v4.0 [33]; clade support was estimated with 1,000 heuristic bootstrap replicates (100 random addition cycles per replicate, with tree bisection-reconnection and branch-swapping [34,35]. For ML analysis, Modeltest 3.06 [36] was used to estimate the appropriate model of DNA substitution for sequence data. The model selected using the Akaike information criterion (AIC) was GTR+I+G. The related parameters of Modeltest were used for ML analysis.

Bayesian phylogenetic analysis and divergence time estimates were done with BEAST 1.5.4 [37,38]. The uncorrelated lognormal relaxed clock model with a Yule process for the speciation model, and GTR+I+G for the substitution model (estimated from the data set) were used. A Markov chain Monte Carlo was run for 50 million generations and sampled every 1,000 generations. Two independent runs were performed to confirm convergence of the analysis. The stationarity of each run was examined using the effective sampling size of each parameter (>200). The last 45 million generations were used to construct the maximum clade credibility tree and associated 95% highest posterior density distributions around the estimated node ages using the program TreeAnnotator 1.5.4, and the tree was visualized using FigTree 1.3.1.

### Constraints

Among angiosperm phylogenetic dating results, the Lamiales clade group has been dated variably as ca. 61.5 Ma [39], ca. 77 Ma [40], ca. 87 Ma [41], ca. 97 Ma [42], and ca. 100~97.5 Ma [43]. A possible balanced age could be ca. 90 Ma, which is the value used in this paper.

Like the Lamiales, the Lamiaceae clade is variably dated by different authors, such as ca. 36.5 Ma by Tank et al.[39], ca. 42~44 Ma by Wikstrom et al. [41], and 58.52 Ma by Magallon et al.[40]. We have chosen 60 Ma, yet considering Lamiaceae tribe Mentheae 37.9~53.8 Ma, and subfamily Nepetoideae ca. 49 Ma [44], probably the 36 to 42~44 Ma times are rather small.

Employing Eocene hexacolpate fossil pollen data, such as species of *Ocimum* L. in Nepetoideae [45], as reviewed by Harley et al. [6] and Drew and Sytsma [44], the pollen fossil of Nepetoideae was constrained as 49 Ma [44]. Reliable fossils fruit of *Melissa* from the Early-Mid Oligocene [46,47] let the MRCA of *Melissa* and *Lepechinia* be constrained as ca. 28 Ma [44,48], which is accepted here. Based on the oldest reliable lamioid fossils so far identified, described from the Seravallian Age of the Middle Miocene flora of Germany, and belonging to *Stachys laticarpa* (seed/fruit) and *Lamium* sp. (13.8~11.6 Ma, Mai 2001), the *Stachys* clade was constrained at 13.8 Ma as per Roy and Lindquist [8].

Therefore, five constraints are included in the BEAST implementation in this paper, prior to Lamiales with normal mean = 90, sd = 1, Lamiaceae with normal 60 = mean, sd = 1, Nepetoideae with lognormal offset = 49 mean = 2.6, sd = 0.5, MRCA of *Melissa* and *Lepechinia* with lognormal offset = 28.4 mean = 1.5, sd = 0.5; the latter two entirely followed Drew and Sytsma [44]. The *Stachys* lognormal offset = 13.8 mean = 0.8, sd = 0.5 followed Roy and Lindquist [8].

### Diversification rate analysis

We ran a series of diversification rate tests to identify potential shifts. Outgroups were pruned for the analyses. Shifts in diversification rates of *Lagochilus* were investigated by inspecting Lineage-through-time (LTT) plots generated using the R package APE v3.1 [49], for the 1000 randomly selected BEAST trees and for the MCC tree. Birth-death likelihood (BDL) models were used to test the significance of heterogeneity or the consistency of the temporal diversification rate [50] [51]. Model selection was based on the difference in AIC scores between the best-fitting rate-constant and rate-variable models (ΔAICRC). The calculations were performed using LASER 2.3 [50]. We further used TreePar [52] to identify the locations of temporal shifts in diversification rates of *Lagochilus*. TreePar analyses were run with a grid setting of 0.1 million years with both Yule and birth-death processes. Rate shifts were recognized as significant when *p* < 0.05 using the likelihood ratio test. Bayesian analysis of macro-evolutionary mixtures [53] was also used to infer speciation rates across the phylogeny. The analyses were run on 1000 randomly sampled BEAST trees. We ran BAMM for 10 million generations and discarded the first 20% as burn-in after checking for convergence. We used the R package BAMMtools [53] to estimate rate-through-time dynamics and number of evolutionary regime shifts from the posterior sampling.

### Ancestral area reconstruction

#### Biogeographical areas and biomes

Eight biogeographical areas were identified based on the species distribution of *Lagochilus*. 1. Most of the species are montane, thus the mountains were subdivided for areas and biomes. The eight areas were: A: Tianshan Mountains, including northern Altau-Tarbagatai and Sunggar-Kashgar, B: Altai, extending to western Siberia, C: Pamir-Alai, D: Iranian Plateau and montane, E: Hindukush, F: Caucasus, G: Turan lowland desert zone, western Central Asia, H: eastern Central Asia, mainly northwestern China and southwestern Mongolia.

The five biomes included A: alpine and subalpine meadow, B: upper montane, gravelly and stony valley and slope, generally steppe, C: lower montane foothill and hillfront, generally desert, D: steppe, E: desert.

#### Ancestral area and biome reconstructions

To infer vicariance, dispersal, and extinction events, three methods were used: the Bayesian statistic parsimony-based method (S-DIVA) [54], a maximum likelihood-based DEC (dispersal extinction cladogenesis) [55,56], and Bayesian binary MCMC (BBM). The three methods were implemented in RASP (Reconstruct Ancestral State in Phylogenies) version 3.2 beta [57].

**Fig 1:**
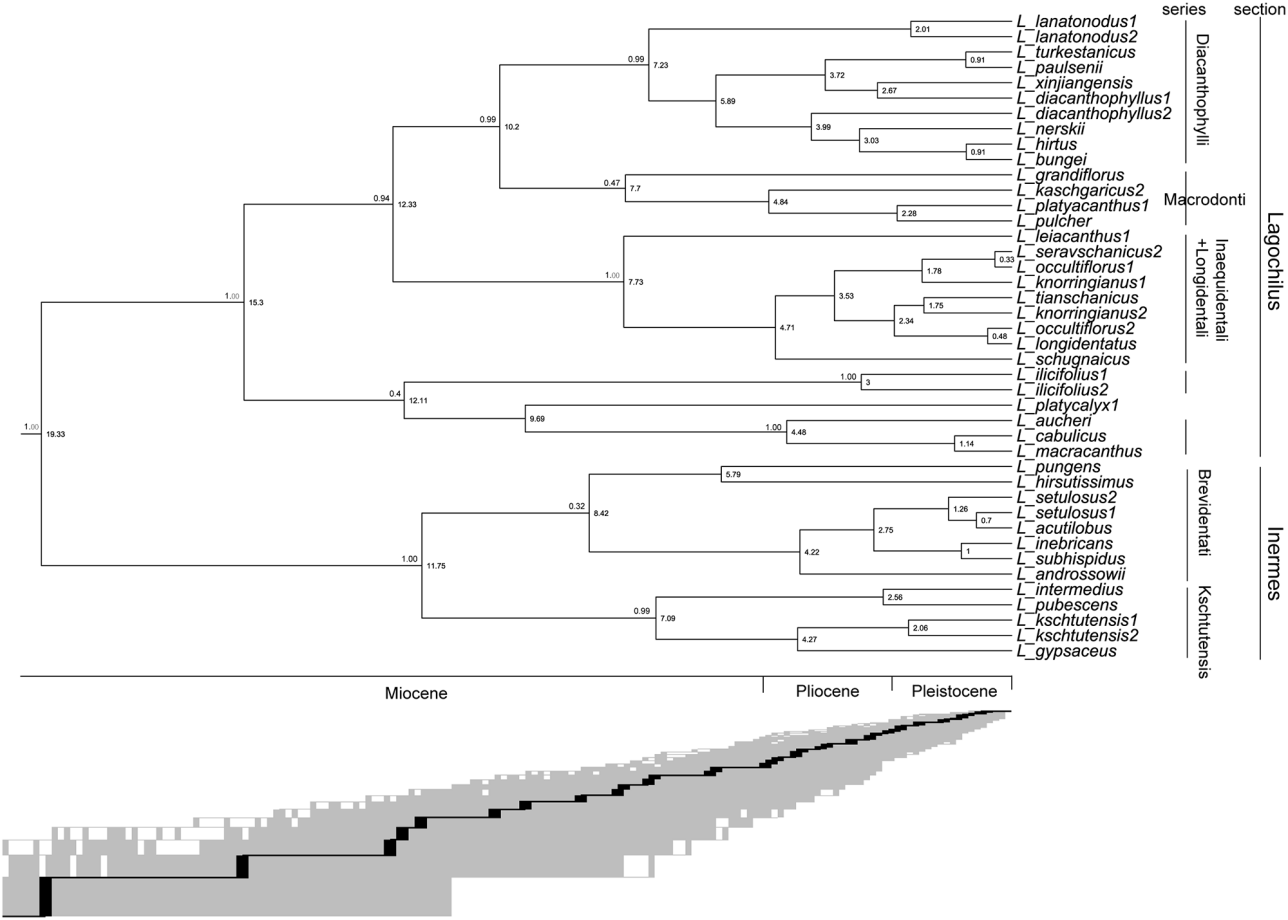
Phylogenetic tree and chronogram using BEAST Bayesian inference. Values at left of nodes on the tree are 95% HPD, at the right are the estimated dating values The classification system derived from tree construction, including two sections and five series, is shown on the right of figure and detailed in the S1 Table. LTT curve illustrated an intimal rapid diversification roughly at 19~12 Ma.

The BEAST molecular dating tree (Fig 1) was treated as a fully resolved phylogram for use as the basis for S-DIVA, with 1000 post-burn-in trees derived from the BEAST analysis used for ancestral area reconstruction in RASP. RASP was performed with various constraints of maximum areas, 2 at each node, to infer possible ancestral areas and potential vicariance and dispersal events. Biogeographical events such as these were calculated under Tree View Form in RASP. DEC [56] was used to calculate the likelihood of biogeographical routes and areas occupied by the most recent common ancestor (MRCA) for the BEAST molecular dating tree (Fig 1) and the present distributions of taxa. Maximum likelihood parameters were estimated for rates of migratory events between areas (range expansions) and local extinctions within areas (range contractions). Like S-DIVA, DEC is used to explore the three most relevant processes of the biogeographical history of a lineage, namely vicariance, dispersal, and extinction.

## Results

### Phylogeny and divergence time estimates

Phylogenetic tree was shown in S1 Fig, which include outgroups and support values, and only *Lagochilus* taxa pruned outgroups was shown in Fig 1. S1 Fig shows that *Lagochilus* is monophyletic with high support (pP = 1.00), and Leonureae as well (pP = 1.00). *Lagochilus* has taxonomically two sections, section *Inermes* is characterized by lacking spinescent bracteoles in the leaf axils of sterile branches, and section *Lagochilus* has spinescent bracteoles. This division was supported roughly by the current tree, except for three species, *L*. *illciflorus* and the clade of *L*. *bungei* and *L*. *hirtus*, to be nested in parts of section *Lagochilus*, but previously they were classified to section *Inermes*.

Six clades were identified within the genus, roughly corresponding to existing taxonomic series [10–12,14], see Fig 1. However, clades of *L*. *ilicifolius*, series *Platycalyces* Knorring (*L*. *platycalyx*), series *Macrodonti* Knorring, and Iran-Afghan clade (*L*. *aucheri*, *L*. *cabulicus*, *L*. *macracanthus*), constituted a new complex, which is not consistent with the existing series rank [12].

The estimated crown age of clade Lamioideae was ca. 44.1 Ma, Leonureae was ca. 23.66 Ma, and *Lagochilus* was ca. 19.33 Ma. This is quite different from the reported dates of Lamioideae ca. 23.9 Ma and Leonureae ca. 9.4~5.7 Ma of Roy and Lindqvist [8]. The diversification ages of section *Inermes* and *Lagochilus* crown clade, were respectively ca. 11.75 Ma and 15.3 Ma. This shows that section’s diversification of the spine and non-spine trait occurred in the middle Miocene. Estimated ages of the clades, roughly, were in a range 7.09~8.42 Ma, except for the special clades of *L*. *ilicifolius* 3 Ma, Iran-Afghan clade (*L*. *aucheri*, *L*. *cabulicus*, *L*. *macracanthus*) was 4.48 Ma.

### Diversification rates

Laser and TreePar analyses did not reject the null hypothesis of a constant diversification rate of *Lagochilus* under both Yule and birth-death processes. For BAMM analyses, the results of rate-through-time dynamics indicate that speciation rates did not increase during its evolutionary history. But The LTT curves of *Lagochilus* plotted as a function of time indicated an initial rapid diversification (Fig 1). Laser and TreePar showed that there was essentially no extinction rate in *Lagochilus*, but a high speciation rate (r = 0.1765 sp/Myr).

### Ancestral area and biome reconstruction

**Fig 2:**
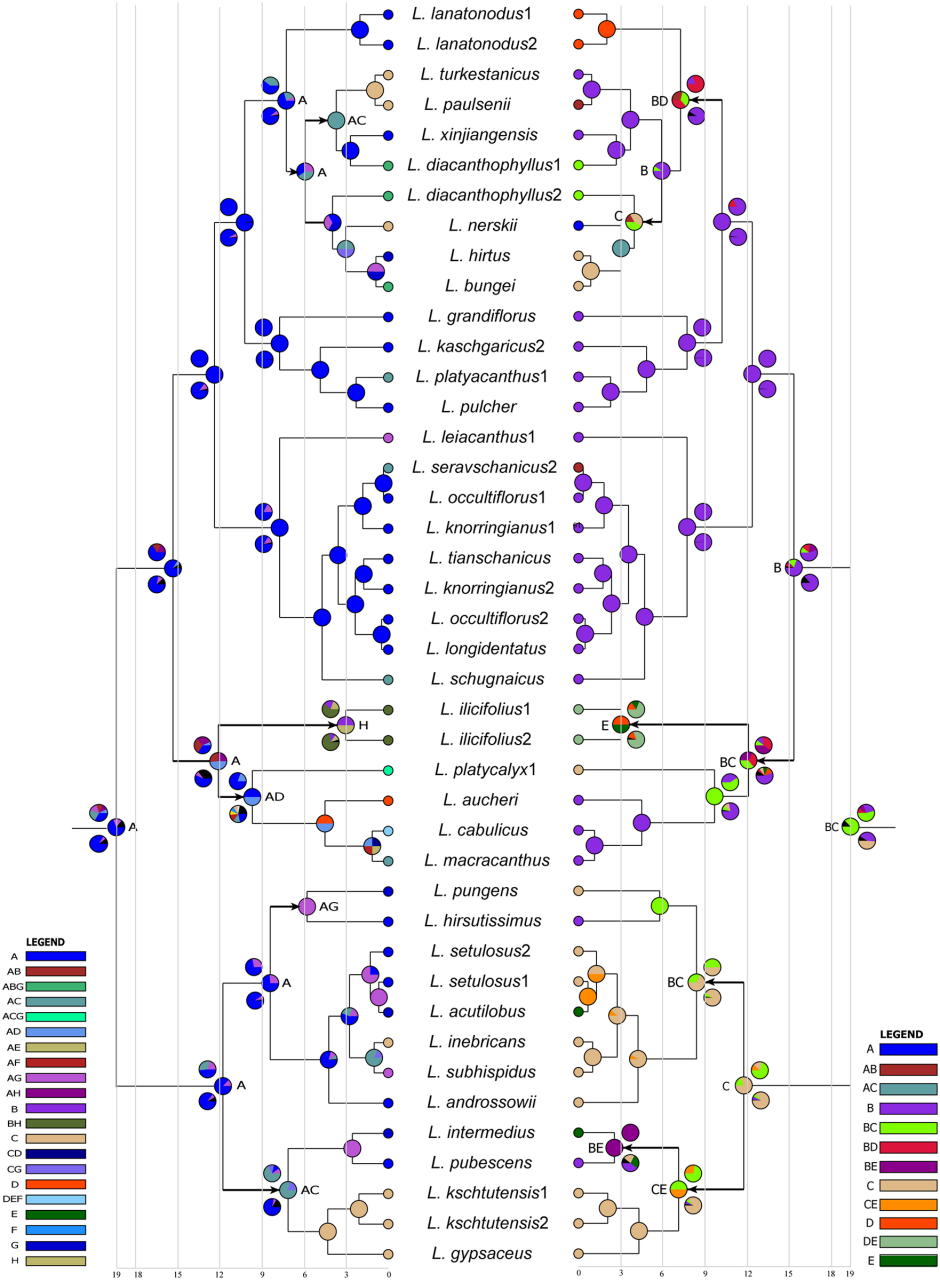
Ancestral area and biome reconstructions, left for area, and right for biome, are performed with approaches of S-DIVA, DEC and BBM using RASP software. Pie charts at nodes of left area are with S-DIVA, left branches above with DEC and below with BBM; at nodes of right biome are with S-DIVA, right branches above with DEC and below with BBM. Several dispersals are shown with arrowheads. Eight operational areas, as stated in text, are: A: Tianshan Mountains, including northern Altau-Tarbagatai, Sunggar-Kashgar, B: Altai, extending to western Siberia, C: Pamir-Alai, D: Iran plateau and montane, E: Hindukush, F: Caucasus, G: Turan lowland desert zone, western Central Asia, H: eastern Central Asia, mainly northwestern China and southwestern Mongolia. Five operational biomes are: A: alpine and subalpine meadow, B: upper montane, gravelly and stony valley, slope, steppe generally, C: lower montane foothill, hillfront, desert generally, D: steppe, E: desert.

In ancestral area reconstruction (Fig 2 left), at the root node of *Lagochilus*, A, Tianshan Mountains, was estimated as clearly the ancestral area by S-DIVA and BBM, whereas DEC indicated A with a lower frequency. At most other nodes, the Tianshan Mountains were also dominant. This indicates that Tianshan Mountains most likely the ancestral area, in where diversification of most species occurred, and several dispersals of other areas, could be regarded as the dispersal results from Tianshan Mountains.

**Fig 3:**
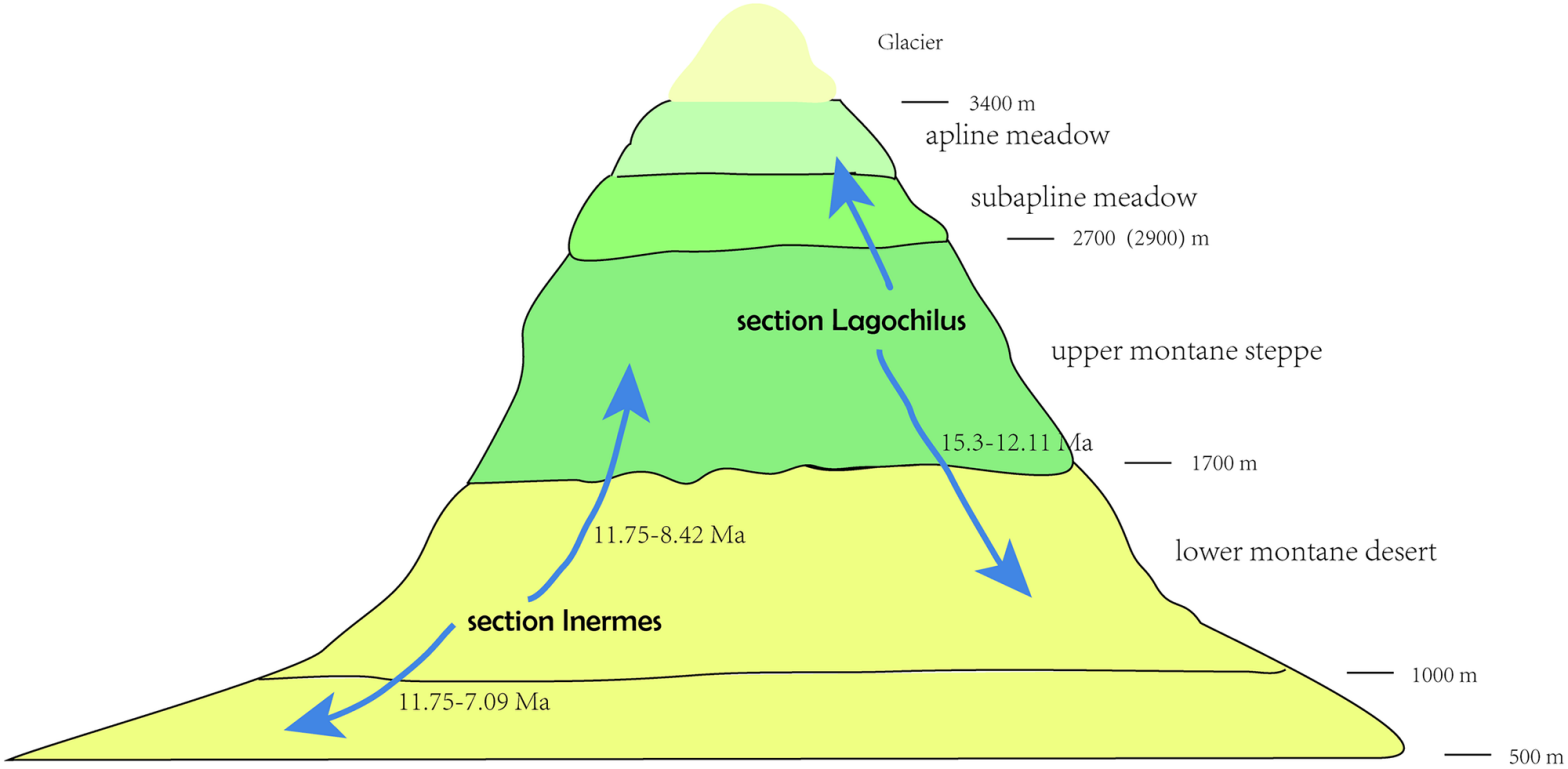
Vertical speciation processes and dispersal events, “downhill” and “uphill” events in two sections respectively, within western Tianshan Mountains in terms of ancestral biome reconstruction, Fig 2.

**Fig 4:**
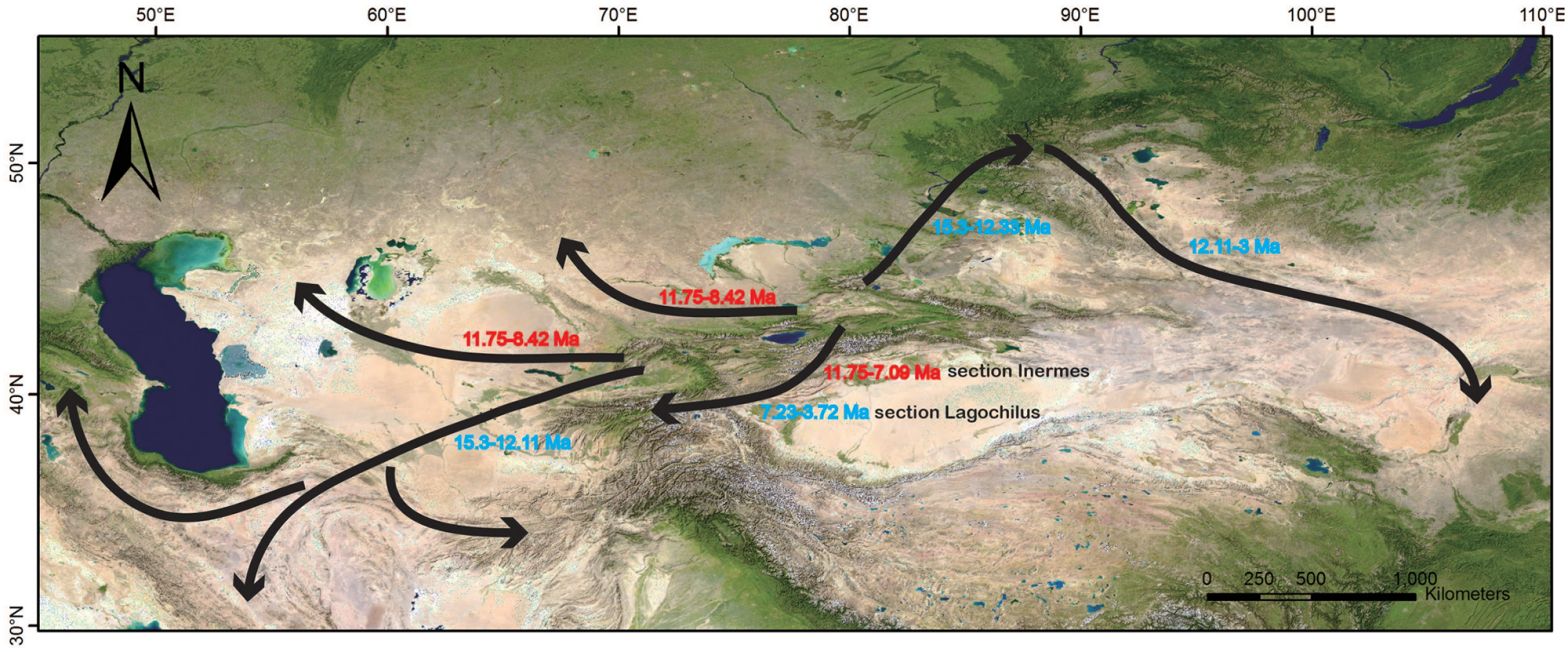
Dispersal events and radiation from western Tianshan Mountains are on the basis of ancestral area reconstructions, Fig 2, the basemap is come from free and public ArcMap 10.0.

Ancestral biome reconstruction (Fig 2 right) showed that a combination of B (upper montane) and C (lower montane) was the ancestral biome of *Lagochilus*. Afterwards, a split appeared in this combination; B formed the ancestral area of section *Lagochilus*, while C was that of section *Inermes*. Due to most species in section *Lagochilus* distributed in biome B, we can consider most diversification of this section to have taken place in the upper montane, similarly, section *Inermes* in lower montane C. Within section *Lagochilus*, there were dispersals from B to C, which we can think as a “downhill” speciation process, whereas within section *Inermes*, those dispersals from C to B, can be thought of as “uphill” speciation, see Fig 3. Species distributed in the biomes of steppe and desert should be regarded as dispersals from montane in the horizontal distribution, see Fig 4.

## Discussion

### Phylogeny and taxonomy

Our molecular evidence revealed that Leonureae is monophyletic, in agreement with Bendiksby et al. [7]. It supported the current classification system of two sections within *Lagochilus*, in terms of the morphological trait whether or not there are spinescent bracteoles at the leaf axils of sterile branches. Exceptionally, however, *L*. *ilicifolius* was nested in section *Lagochilus* rather than section *Inermes* sensu Knorring [12]. After carefully checked the specimens of *L*. *ilicifolius*, we found that many individuals sometimes have spinescent bracteoles in the leaf axils of sterile branches, thus this species should rightly be included in section *Lagochilus* [15]. Meanwhile, Knorring’s [12] subdivision of series ranks of classification was shown to be unjustifiable, mainly, due to many series in section *Lagochilus* being shown to be polyphyletic and reticular in our phylogenetic tree. Our recognized clades in the tree, were approximately equal to several of Knorring’s [12] series but several exclusives of their sectional assignments, especially, *L*. *ilicifolius* clade, series *Platycalyces* Knorring (*L*. *platycalyx*), series *Macrodonti* Knorring, and the Iran-Afghan clade (*L*. *aucheri*, *L*. *cabulicus*, *L*. *macracanthus*) with distinct morphological and geographical attributes. These exclusives are consistent with Zuckerwanik’s [15] classification somewhere, especially her notes of *L*. *ilicifolius* (= subsection *Ilicifolii* Zuckerwanik in section *Inermes* Knorring), series *Platycalyces* Knorring (*L*. *platycalyx*), and series *Macrodonti* Knorring (= subsection *Triangulolobi* Zuckerwanik of section *Lagochilus* Zuckerwanik). In addition, Knorring established the genus *Lagochilopsis* basing on five species *L*. *aculilobus*, *L*. *bungei*, *L*. *hirtus*, *L*. *punngens*, and *L*. *subhispidus* [13], which is probably problematic since five species could not combined to a clade and they nested different clades. Anyway, division of these groups in phylogenetic tree (Fig 1) should be regarded as an improvement of the previous taxonomies and a foundation of further classification.

### Divergence time estimate and morphological variation

Roy and Lindqvist [8] dated the crown age of subfamily Lamioideae as ca. 23.9 Ma and tribe Leonureae ca. 9.4~5.7 Ma, which probably resulted from a small constraint value for Lamiaceae of 28.4 Ma [8,47], accordingly, these two estimated ages values were rather low. Based on the current results of angiosperm molecular dating [39–42], our constraint values for Lamiales and Lamiaceae etc. in this paper, resulted in a larger estimated age of ca. 44.1 Ma for Lamioideae.

Compared with the estimated ages of subfamily Nepteoideae ca. 56 Ma, as well its three tribes Mentheae ca. 46 Ma, Ocimeae ca. 43 Ma and Elsholtzieae ca. 34 Ma yielded by Drew and Sytsma [18], the estimated ages of Lamioideae and its members ca. 23.9 Ma by Roy and Lindqvist [8] are probably too small, whereas our estimated age of ca. 44.1 Ma for Lamioideae seems preferable.

There is no the crown age of *Lagochilus* from Roy and Lindqvist [8], since only one species, *Lagochilus ilicifolius*, was sampled. However, undoubtedly, *Lagochilus* could be hypothesized young in late Miocene behind their estimated age of Leonureae ca. 9.4~5.7 Ma. Looking back the estimated ages of Central Asian plant genera, such as *Caragana* ca. 16.15 Ma [58], *Myricaria* ca. 20.25 Ma [23], *Reamuria* ca. 32.95 Ma [59] and *Atraphaxis* ca. 26.67 Ma [60], Asian *Zygophllum* ca. 19.56 Ma [61], *Fagonia* ca. 20.19 Ma [61], *Ephedra* ca. 27.85 Ma [62], all with ages of at least early Miocene or more ancient, our estimated diversification age of the crown clade of ca. 19.33 Ma for *Lagochilus* would be justifiable. Combined with a stem age of ca. 23.66 Ma of *Lagochilus*, we can suggest that the time of origin of the genus should be late Oligocene to early Miocene approximately 23.66~19.33 Ma. As a typical automorphy of *Lagochilus* different from other genera in Leonureae, the trait of spinescent bracteoles should have been formed at this time of origin.

Whereas morphological diversifications of the two sections, i.e. whether or not with spinescent bracteoles at leaf axils of sterile branches [10,12], might date to middle Miocene, 15.3 Ma for section *Lagochilus* and 11.75 Ma for section *Inermes* respectively, by our molecular dating scheme. LTT curve of diversification rates analysis shown a rapid divergence during 19~12 Ma in Fig 1, it is consistent with generic and sectional diversification of morphological diversification.

The estimated crown ages of the clades, roughly corresponding to the ages of Knorring’s [12] series ranks, falled into the range of late Miocene 8.42~7.09 Ma. These series are distinguished mainly by morphological variation of calyx teeth number, shape, and size [12], which accordingly should diversified at this period.

The clade at node ca. 12.11 Ma in Fig 1 is comprised young “series” clades, *L*. *ilicifolius* is distributed in Altai—Gobi Altai—eastern Mongolian Plateau with ca. 3 Ma, and series *Macrodonti* Knorring in Iran-Afghan is ca. 4.48 Ma. Series *Platycalyces* Knorring (*L*. *platycalyx*) widespread in Tianshan, Pamir—Alai, and Turan, has a diversified age ca. 9.69 Ma.

### Tianshan montane origin, diversification and radiation

The results of ancestral area reconstruction indicated clearly that the Tianshan Mountains is the ancestral area. Since most species occur in the Ili-Kirghizia Tianshan, as well as Sunggar and Kaschgar (see S1 Table), the western Tianshan Mountains should be the diversification center.

As mentioned above, diversification of two sections and formation of the spiny attribute took place in the Tianshan Mountains, from where, dispersals in section *Inermes* were mainly southward to the Pamir-Alai at ca. 12~7 Ma, westward to the Turan lowland desert zone, including the Balkhash District, Caspian-Aral Sea, Kyzalkum, and Karakum at ca. 12~8 Ma. In section *Lagochilus* were northward to Sunggar-Karatau, Tarbagatai, and Altai at ca. 15~12 Ma, then eastward to the Gobi-Altai and Mongolian Plateau, Helanshan Mts. and the Loess Plateau, mainly along the *L*. *ilicifolius* distribution ca. (12) to 3 Ma, southward Pamir-Alai ca. 7.23~3.72 Ma, southwestward to the Iranian Plateau ca. 15~12 Ma, then to the Caucasus and Hindukush, outlined in Fig 4. On the whole, all dispersals are from the western Tianshan Mountains to surrounding areas, or all dispersals could be also regarded as the radiations, and happened in middle-late Miocene.

Within Tianshan Mountains, the ancestral distribution of species biomes was shown to be in the montane steppe zone of valley and slope, and the desert zone of foothill and front-hill. In terms of trait of the western Tianshan Mountains [63,64], these montane distribution zones of *Lagochilus* species are generally in gravelly and stony montane steppe and desert, approximately at altitudes of 1700–2700 m in steppe and 1000–1700 m in desert.

Coupled with diversification of the two sections *Inermes* and *Lagochilus* in *Lagochilus*, the ancestral montane biome was divided into two parts, namely, the montane steppe zone of valley and slope with section *Lagochilus*, and the desert zone in foothill and front-hill with section *Inermes*. The spiny morphological character developed in the upper montane but not the lower desert zone, so it might be a defense to grazing animals in the more favorable upper zone. Possibly, therefore, *L*.*illicifolius*, having dispersed out of that zone, no longer experienced the same adaptive pressure for spine, and had begun to lose that trait. In section*Lagochilus*, the speciation process is generally “downhill” from the montane steppe zone to the montane desert zone, whereas in section *Inermes* it is “uphill” from montane desert to montane steppe. Distribution of a few species in the subalpine zone in section *Lagochilus* is “uphill” from valley and slope of montane steppe. Whether “downhill” or “uphill”, the speciation process of dispersals within the montane zones in Miocene times is explainable since species exchanging between montane steppe and desert in late Miocene is no-barrier [62,62], described in Fig 4.

The origin of Central Asian flora is a complicated issue, there are many different or controversial hypotheses (see reviews [21,65]), such as the native [66–68], Mediterranean or African [21,69,70]. *Atraphaxis* was regarded as a typical case of origination from Tianshan Mountains to Central Asian flora. *Lagochilus* with origin and diversification of Tianshan Mountains, contributes another case and enriches the hypotheses of origin of Central Asian flora. At the time-dimension, Popov [71] proposed three evolutionary stages for Central Asian flora, i.e. from Cretaceous to Early Tertiary, Later Tertiary, and from the Later Tertiary onward. *Lagochilus* origin age ca. 23.66~19.33 Ma should be seated at Later Tertiary. Its origin during late Oligocene to early Miocene, just likes a series of lineages of Central Asian flora, such as the mentioned *Caragana*, *Myricaria*, *Reamuria*, *Atraphaxis*, *Zygophllum*, *Fagonia* and *Ephedra*.

### Dynamics of montane origin and diversification

To explain the *Lagochilus* spatiotemporal origin and diversification described above, we link temporal arid paleogeographical and paleoclimate events in Central Asia. Even though *Lagochilus* has no species in the QTP or Himalayas, however, the Pamir-Alai and Tianshan mountains and northwestern China are located on the north of the QTP and Himalayas, particularly, Tianshan uplift is inferred to have been associated with QTP uplift [63]. Asian interior aridification is hypothesized to have been affected remarkably by QTP uplift [64,72]. Therefore, we need trace back to QTP uplift. In general, it is hypothesized to have three stages [64,72], at first, collision of the Indian and Eurasian continents at ca. 50 Ma; second, the Himalayan Motion at about 25~17 Ma, and third, intense uplift starting at 3.6 Ma. Whilst Tianshan uplift, has two stages, the ancient uplift was during late Jurassic to early Cretaceous, and the rapid uplift interval was from Oligocene to Miocene 25~16 Ma [63,73,74]. Therefore, Tianshan uplift in Cenozoic was synchronized with the Himalayan Motion and to have been regarded as its distant influence [63].

Accordingly, origin and diversification of *Lagochilus* during the Oligocene to Miocene 23.66~19.33 Ma in western Tianshan montane, can be thought as driven by Tianshan uplift as well as Himalayan Motion at ca. 25~16 Ma. Also, the origin and diversification was in montane valley, slope, and foothill and hillfront. Tianshan uplift also resulted in origin of the attribute of spinescent bracteoles, and expansion of distribution ranges, see Figs 1–3. Many cases have accumulated concerning the origin and diversification of arid plant lineages driven by the Himalayan Motion, such as our recent studies of *Caragana* [58] and *Myricaria* [23], but direct linkage of Tianshan uplift and the arid lineage diversification is lacking. *Lagochilus* contributes a case.

Further diversifications of the genus, spiny traits emerging of section *Lagochilus* at ca. 15.3 Ma, falls into the interval of Central Asian climate aridification 17~5 Ma, which is hypothesized to have resulted from QTP uplift [64,75–77]. Many other morphological and phylogenetic responses to this aridification process can be cited by species dispersals and radiation from western Tianshan montane (Fig 4); “downhill” and “uphill” events within the montane biome zone (Fig 3) etc. The principal QTP uplift in 13~7 Ma [78,79], and global cooling and aridification at 8~7 Ma [80], should be linked the various calyx teeth variations at series rank or clades of the genus (ca. 7.23, 7.7, 7.73, 8.42, 7.09 Ma, Fig 1). Even though many previous studies have treated the spines of leaves, stems, or bracts as an adaptation of preserving water to arid climate, such as in *Cactus* (Cactaceae), or in the Xinjiang desert zone of China, *Alhagi sparsifolia* Shap and, *Caragana pleiophylla* (Regel) Pojarkova (Fabaceae), and *Convolvulus tragacanthoides* Turcz. (Convolvulaceae), however, there is no a genus case with spiny morphological variation to document climatic action like *Lagochilus* in this paper. On the whole, arid *Lagochilus* with a distribution pattern of montane steppe and desert in the Tianshan, Sunggar-Kaschgar, Turan area, and with spiny morphological character variation, most likely resulted from the arid and cooling paleoclimate during middle-late Miocene.

## Supporting information

S1 Table Voucher information for sequenced *Lagochilus* and outgroups. (DOC)

S2 Table Sequence data of outgroups downloaded from the GenBank. (DOC)

S1 Fig Phylogenetic tree of *Lagochilus* and outgroups and 95% posterior probablity support at the nodes using BEAST. (TIF)
