# Supplementary material for: Insight into Central Asian flora from the Cenozoic Tianshan montane origin and radiation of Lagochilus (Lamiaceae)
Source: PLoS One. 2017 Sep 20;12(9):e0178389. doi: 10.1371/journal.pone.0178389 (PMC5606930; doi:10.1371/journal.pone.0178389)
Supplement: S1 Table — (DOC) [file pone.0178389.s001.doc]

| Taxon | Voucher | Source | *rps16* | *psb*A-*trn*H | *mat*K | *trn*L-trnF | *psb*B-*psb*H | *psb*K-*psb*I | Area | Biome |
| --- | --- | --- | --- | --- | --- | --- | --- | --- | --- | --- |
| ***Lagochilus* Bunge** |  |  |  | | | | | |  |  |
| *L. aculilobus* (Lad.) Fisch. et Mey. | Darbaeva T.E. 13 VIII 2001 (LE) | NW Kazakhstan, Gurevskaya Prov., Emba Plato | MF157179 | MF157023 | MF156975 | MF157231 | MF157075 | MF157127 | G | E |
| *L. androssovii* Knorr. | A. Yunatov, L. Kuznetsov11 VI 1956 (LE) | Kazakhstan, Kyzyl-Ordinskaya Prov., foothills of Ridge Karatau | MF157180 | MF157024 | MF156976 | MF157232 | MF157076 | MF157128 | A | C |
| *L. aucheri* Boiss. | Wendelbo & Assadi 13257 (MHA) | Iran, Masandaran, Lar valley | MF157181 | MF157025 | MF156977 | MF157233 | MF157077 | MF157129 | D | B |
| *L. bungei* Benth. | Sh. Dariimaa, R.V. Kamelin 224 7 VI 1984 (LE) | Mongolia, Mongolian Altai, valley of Rv. Bulgan-gol | MF157182 | MF157026 | MF156978 | MF157234 | MF157078 | MF157130 | ABG | C |
| *L. cabulicus* Benth. | Khandjan N.S., Manakjan s.n. 6 VI 1985, (LE) | Iran, Nakhichevan, Ordubadsky distr. | MF157183 | MF157027 | MF156979 | MF157235 | MF157079 | MF157131 | DEF | B |
| *L. diacanthophyllus* Benth. 1 | Sh. Dariimaa, R.V. Kamelin 531 16 VI 1984 (LE) | Mongolia, Mongolian Altai, valley of Rv. Bulgangol | MF157184 | MF157028 | MF156980 | MF157236 | MF157080 | MF157132 | ABG | BC |
| *L. diacanthophyllus* Benth.2 | Meng H.H. L322 (XJBI) | China, Xinjiang, Altai | MF157185 | MF157029 | MF156981 | MF157237 | MF157081 | MF157133 | ABC | BC |
| *L. grandiflorus* C.Y. Wu & S.J. Hsuan | Meng H.H. L267(XJBI) | China, Xinjiang, Tekes | MF157186 | MF157030 | MF156982 | MF157238 | MF157082 | MF157134 | ABG | BC |
| *L. gypsaceus* Vved. | V. Bochantsev13 V 1975 (LE) | S Kazakhstan, Mountains Aruk | MF157187 | MF157031 | MF156983 | MF157239 | MF157083 | MF157135 | A | B |
| *L. hirsutissimus* Vved. | Pratov, Tsukervanik, Makhmedov 764 28 V 1976 (LE) | W. Tianshan, Ridge Kuraminsky, Kamchik pass | MF157188 | MF157032 | MF156023 | MF157240 | MF157084 | MF157136 | C | C |
| *L. hirtus* Fisch. et Mey. | Meng H.H. L213 (XJBI) | China, Xinjiang, Hefeng | MF157189 | MF157033 | MF156024 | MF157241 | MF157085 | MF157137 | A | B |
| *L. ilicifolius* Bunge ex Benth. 1 | S. Timokhina, L. Danilyuk 1339 1 VIII 1973 (LE) | Tuva, Ovyursky distr. | MF157190 | MF157034 | …………. | …………. | MF157086 | MF157138 | G | C |
| *L. ilicifolius* Bunge ex Benth.2 | I.A. Gubanov 253, 2 VIII 1989 (MW) | Mongolia, S Gobi Aimak, Alashan Gobi | MF157191 | MF157035 | MF156984 | MF157242 | MF157087 | MF157139 | C | C |
| *L. inebricans* Bunge | Prokuryakova s.n. 7 VI 1970 (MHA) | Turcomania, red colored ground near vil. Charshangu | MF157192 | MF157036 | MF156985 | MF157243 | MF157088 | MF157140 | BH | DE |
| *L. intermedius* Vved. | N.A. Kazaekbaev 2 VI 1967 (LE) | Uzbekistan, desert Kysylkun | MF157193 | MF157037 | MF156986 | MF157244 | MF157089 | MF157141 | BH | DE |
| *L. kaschgaricus* Rupr.1 | Milko D.A. 26 VII 2009 (LE) | Kyrghyzstan, Narynskay Prov., Aktalinsky distr. | MF157194 | MF157038 | MF156987 | MF157245 | MF157090 | MF157142 | C | C |
| *L. kaschgaricus* Rupr.2 | Meng H.H. L309 (XJBI) | China, Xinjiang, Kashi | MF157195 | MF157039 | MF156988 | MF157246 | MF157091 | MF157143 |  |  |
| *L. knorringianus* Pavlov1 | Pratov, Tsukervanik, Makhmedov 696 24 V 1976 (LE) | Tadzhikistan, Pamir-Alai, Ridge Turkestansky ridge | MF157196 | MF157040 | MF156989 | MF157247 | MF157092 | MF157144 | G | E |
| *L. knorringianus* Pavlov2 | M.P. Piemenov et al. K96-151, 12 VI 1996 (MW) | Kyrghyzstan, Andijon | MF157197 | MF157041 | MF156990 | MF157248 | MF157093 | MF157145 | G | E |
| *L. kschtutensis* Boiss.1 | R.V. Kamelin 497, 6 IV 1979 (LE) | Tadzhikistan, Zaravshansky Ridge | MF157198 | MF157042 | MF156991 | MF157249 | MF157094 | MF157146 | A | B |
| *L. kschtutensis* Boiss.2 | M.G. Pimenov, E.V. Klyuikov 137, 15 VI 1990 (MW), | Tadzhikistan, Leninabad, N slope of Zaravshan Ridge | MF157199 | MF157043 | MF156992 | MF157250 | MF157095 | MF157147 | A | B |
| *L. lanatonodus* C.Y. Wu et S.J.Hsuan1 | Meng H.H. L209 (XJBI) | China, Xinjiang, Yiwu | MF157200 | MF157044 | MF156993 | MF157251 | MF157096 | MF157148 | A | B |
| *L. lanatonodus* C.Y. Wu et S.J.Hsuan2 | Meng H.H. L254 (XJBI) | China, Xinjiang, Sailimu Lake side | MF157201 | MF157045 | MF156994 | MF157252 | MF157097 | MF157149 | A | B |
| *L. leiacanthus* Fisch. et Mey.1 | R.V. Kamelin 1264, 10 VI 1974 (LE) | Kazakhstan, Mountains Ulokunburug | MF157202 | MF157046 | MF156995 | MF157253 | MF157098 | MF157150 | A | B |
| *L. leiacanthus* Fisch. et Mey.2 | V.P. Goloskokov, 2 VII 1956 (MW) | Kazakhstan, S ridges of Dzhungar Alatau, Mountains Katutau | MF157203 | MF157047 | MF156996 | MF157254 | MF157099 | MF157151 | A | B |
| *L. longidentatus* Knorr. | M.G. Pimenov, M.G. Vassiljeva, S. Daushkevich 159 18 VIII 1982 (MW) | S. Kazakhstan, Ridge Syrdarinsky Karatu, slopes of Mts. Mindzhilke | MF157204 | MF157048 | …………. | …………. | MF157100 | MF157152 | C | C |
| *L. macracantus* Fisch. et Mey. | Wendelbo, Shirdelpur, Assadi 12531 (MHA) | Iran, Tehran, Vardavard valley | MF157205 | MF157049 | MF156997 | MF157255 | MF157101 | MF157153 | C | C |
| *L. nerskii* Knorr. | Vasilchenko, Sabirov, Ismailova 227, 18 VIII 1958 (MW) | Baisunskie [Baisun] mountains, upward on Rv. Irgaili-sai | MF157206 | MF157050 | MF156998 | MF157256 | MF157102 | MF157154 | A | D |
| *L. occultiflorus* Rupr.1 | M.G. Pimenov, E.V. Kljuykov n. 171, 3 VII 2000 (MW) | S. Kazakhstan, Chimkentskaya Prov., Ugamsky Ridge | MF157207 | MF157051 | MF156999 | MF157257 | MF157103 | MF157155 | A | D |
| *L. occultiflorus* Rupr.2 | M.G. Pimenov K95-190, 6 VIII 1995 (MW) | Kirgizstan, Kishtebo | MF157208 | MF157052 | MF157000 | MF157258 | MF157104 | MF157156 | A | D |
| *L. paulsenii* Briq. | Makhmedov A.M. 523, 14 VII 1979 (LE) | Kazakhstan, Pamir-Alai, Alaisky Ridge | MF157209 | MF157053 | MF157001 | MF157259 | MF157105 | MF157157 | AG | B |
| *L. platyacanthus* Rupr.1 | Bochantsev V.P., Aidarova R.A. s.n. 6 VIII 1979 (LE) | Kirgizstan, Rv. Kara-Kudzhur, right side | MF157210 | MF157054 | MF157002 | MF157260 | MF157106 | MF157158 | A | B |
| *L. platyacanthus* Rupr.2 | Meng H.H. L288 (XJBI) | China, Xinjiang, Wuchia | MF157211 | MF157055 | MF157003 | MF157261 | MF157107 | MF157159 | A | B |
| *L. platycalyx* Fisch. et Mey.1 | I.I. Mikhova N.D. Tokmachjova 788, 6 VI 1987 (LE) | Kazakhstan, Chimkentskaya Prov. Tyul’kubassky distr. | MF157212 | MF157056 | MF157004 | MF157262 | MF157108 | MF157160 | AC | B |
| *L. platycalyx* Fisch. et Mey.2 | I.I. Rusanovich, 9 VI 1987 (MHA) | Uzbekistan, Chimkent, Karatau Ridge, Turan pass | MF157213 | MF157057 | MF157005 | MF157263 | MF157109 | MF157161 | AC | B |
| *L. pubescens* Vved. | T. Tsukervanik 5 VII 1971 (LE) | Tadzhikistan, Pamir-Alai, Ridge Alaisky ridge | MF157214 | MF157058 | MF157006 | MF157264 | MF157110 | MF157162 | C | A |
| *L. pulcher* Knorr. | Taishanov, Nigmatullaev 5651, 30 V 1980 (LE) | Uzbekistan, Ridge Ketmen, foothills | MF157215 | MF157059 | MF157007 | MF157265 | MF157111 | MF157163 | A | B |
| *L. pungens* Schrenk | Taishanov, Nigmatullaev 5675, 4 VI 1980 (LE) | Uzbekistan, N foothills of Ridge Ketmen lower part of Rv. Charyn | MF157216 | MF157060 | MF157008 | MF157266 | MF157112 | MF157164 | A | B |
| *L. schugnanicus* Knorr. | G.M. Ladygin, Rachkovskaya 3169, 17 VIII 1981 (LE) | Kirgizstan, S macro slope of Alaisky Ridge | MF157217 | MF157061 | MF157009 | MF157267 | MF157113 | MF157165 | C | AB |
| *L. seravschanicus* Knorr. | P. Uotila 47785, 09 VIII, 2009 (MW) | Kirghizia,Jabal-Abad Region, Chatkal District | MF157218 | MF157062 | MF157010 | MF157268 | MF157114 | MF157166 | AC | B |
| *L. seravschanicus* Knorr.2 | N. Sheveliova, T. Konovalova, 19 VII 1993 (MHA) | Tajikistan, W Pamir, E macro-slope of Vakhang Ridge, opposite of Horog botanical garden | MF157219 | MF157063 | MF157011 | MF157269 | MF157115 | MF157167 | AC | B |
| *L. setulosus* Vved. | R.V. Kamelin 16, 22 VI 1973 (LE) | S Kazakhstan, Mountains Alym-tau | MF157220 | MF157064 | MF157012 | MF157270 | MF157116 | MF157168 | ACG | C |
| *L. setulosus* Vved. | N.P. Khokhryakov, 29 VIII 1960 (MHA) | Kazakhstan, environs of station Darbasa | MF157221 | MF157065 | MF157013 | MF157271 | MF157117 | MF157169 | ACG | C |
| *L. subhispidus* Knorr. | R. Kamelin 847a 25 VII 1970 (LE) | Kirghizia, Talasskaya valley, left bank of Rv. Talass | MF157222 | MF157066 | MF157014 | MF157272 | MF157118 | MF157170 | A | B |
| *L. tianschanicus* Pavlov | V. Pavlov,10 VIII 1966 (MW) | W Tianshan, Talassky Alatau, Reserve Aksu-Dzebagly, gorge Kish-Kaindy on SE slope, 2000 m alt. | MF157224 | MF157068 | MF157016 | MF157274 | MF157120 | MF157172 | A | B |
| *L. turkestanicus* Knorr. | Miryakin [?] s.n. 12 X 1957(LE) | Kirgizia, Loilyaksky distr. | MF157223 | MF157067 | MF157015 | MF157273 | MF157119 | MF157171 | G | C |
| *L. xinjiangensis* G.J. Liu | Meng H.H. L314 (XJBI) | China, Xinjiang, Urumqi | MF157225 | MF157069 | MF157017 | MF157275 | MF157121 | MF157173 | AC | B |
| ***Panzenia* Moench** |  |  |  |  |  |  |  |  |  |  |
| *P. canescens* Bunge 1 | Kamelin R.V., Shmakov A. et al. 11 July 1992 (LE) | Republica of Altai, Kosh Agachsky distr., valley of Rv. Tshuya | MF157226 | MF157070 | MF157018 | MF157276 | MF157122 | MF157174 |  |  |
| *P. canescens* Bunge 2 | I.A. Gubanov, R.V. Kamelin n. 1001, 9 VII 1988 (MW) | Mongolia, Altai, 40 km to NW from vil. Ulan-Khus of Bayan-Ulehgehei aimak | MF157227 | MF157071 | MF157019 | MF157277 | MF157123 | MF157175 |  |  |
| *P. lanata* Bunge | I. Pshenichnaya, G. Liventseva s.n. 23 III 1985 (LE, NS) | Mongolia, Altai, Shabalinsky distr., Seminsky Ridge | MF157228 | MF157072 | MF157020 | MF157278 | MF157124 | MF157176 |  |  |
| *P. minor* | Meng H.H. L326 (XJBI) | China, Xinjiang, Tekes | MF157229 | MF157073 | MF157021 | MF157279 | MF157125 | MF157177 |  |  |
| ***Leonurus* L.** |  |  |  |  |  |  |  |  |  |  |
| *L. glaucescens* Bunge | Akhmetzhanova A.A. 23 VII 2010 (MW) | Kazakhstan, Western Kazakhskaya (Ural’skaya) Prov., Teretinsky distr. | MF157230 | MF157074 | MF157022 | MF157280 | MF157126 | MF157178 |  |  |

Table S1. Voucher information for sequenced *Lagochilus* and outgroups. A: Tianshan Mountains, including northern Altau-Tarhabatai, Sunggar-Kashgar, B: Altai, extending western Siberia, C: Pamir-Alai, D: Iran plateau and montane, E: Hindukush, F: Caucasus, G: Turan lowland desert zone, western Central Asia, H: eastern Central Asia, mainly northwestern China and southwestern Mongolia. Five biomes included A: alpine and subalpine meadow, B: upper montane, gravelly and stony valley, slope, steppe generally, C: lower montane foothill, hillfront, generally desert, D: steppe, E: desert.

Herbaria LE: Komarov Botanical Institute, Russian Academy of Sciences (St. Petersburg), MW: Moscow University (Moscow), MHA: Main Botanical Garden, Russian Academy of Sciences (Moscow), and XIBJ: Xinjiang Institute of Ecology and Geography, Chinese Academy of Sciences (Urumqi).
