# Supplementary material for: Insight into Central Asian flora from the Cenozoic Tianshan montane origin and radiation of Lagochilus (Lamiaceae)
Source: PLoS One. 2017 Sep 20;12(9):e0178389. doi: 10.1371/journal.pone.0178389 (PMC5606930; doi:10.1371/journal.pone.0178389)
Supplement: S2 Table — (DOC) [file pone.0178389.s002.doc]

S2 Table. Sequence data downloaded from the GenBank.

***mat*K**—*Acanthus longifolius* Host, AJ429326; [*Achyrospermum africanum*](http://www.ncbi.nlm.nih.gov/nuccore/FJ854133.1) [Hook.f.](http://www.ipni.org/ipni/idAuthorSearch.do;jsessionid=44E655B352D5E518E7C1EB569476281B?id=4084-1&back_page=/ipni/editSimplePlantNameSearch.do;jsessionid=807A82803C08AAD4D2513DE7839BA78D?find_wholeName=Achyrospermum+africanum&output_format=normal) ex [Baker](http://www.ipni.org/ipni/idAuthorSearch.do;jsessionid=44E655B352D5E518E7C1EB569476281B?id=407-1&back_page=/ipni/editSimplePlantNameSearch.do;jsessionid=807A82803C08AAD4D2513DE7839BA78D?find_wholeName=Achyrospermum+africanum&output_format=normal), HQ911418; *Elsholtzia stauntonii* [Benth.](http://www.ipni.org/ipni/idPlantNameSearch.do?id=446697-1&back_page=/ipni/editSimplePlantNameSearch.do?find_wholeName=Elsholtzia+stauntonii&output_format=normal), KP089058; *Hemigenia pedunculata* [Diels](http://www.ipni.org/ipni/idPlantNameSearch.do?id=447764-1&back_page=/ipni/editSimplePlantNameSearch.do?find_wholeName=Hemigenia+pedunculata&output_format=normal), HQ911374;

*Lamium galeobdolon* var. montanum, JF779870; *Lamium tomentosum* [Benth. ex Nyman](http://www.ipni.org/ipni/idPlantNameSearch.do?id=448965-1&back_page=/ipni/editSimplePlantNameSearch.do?find_wholeName=Lamium+tomentosum&output_format=normal), HQ911459; *Melissa officinalis* L., KJ196364; *Olea europaea* L., AJ429335; *Origanum vulgare* L., KJ204514; *Physostegia ledinghamii* [(Boivin) P.D.Cantino](http://www.ipni.org/ipni/idPlantNameSearch.do?id=909563-1&back_page=/ipni/editSimplePlantNameSearch.do?find_wholeName=Physostegia+ledinghamii&output_format=normal), HQ911435; *Physostegia pulchella* [Lundell](http://www.ipni.org/ipni/idPlantNameSearch.do?id=454195-1&back_page=/ipni/editSimplePlantNameSearch.do?find_wholeName=Physostegia+pulchella&output_format=normal), HQ911440; *Stachys nephrophylla* [Rech.f.](http://www.ipni.org/ipni/idPlantNameSearch.do?id=459783-1&back_page=/ipni/editSimplePlantNameSearch.do?find_wholeName=Stachys+nephrophylla&output_format=normal), HQ911548; *Stachys spinosa* L., HQ911537;

*Thymus serpyllum* L., JF357823.

***psb*A-**trn**H**— *Elsholtzia stauntonii* [Benth.](http://www.ipni.org/ipni/idPlantNameSearch.do?id=446697-1&back_page=/ipni/editSimplePlantNameSearch.do?find_wholeName=Elsholtzia+stauntonii&output_format=normal), FJ513098; *Lamium galeobdolon* var. montanum, JF780149; *Lamium tomentosum* [Benth. ex Nyman](http://www.ipni.org/ipni/idPlantNameSearch.do?id=448965-1&back_page=/ipni/editSimplePlantNameSearch.do?find_wholeName=Lamium+tomentosum&output_format=normal), JF780186; *Lepechinia calycina* Epl, DQ667394;. *Melissa officinalis* L., DQ667387; *Olea europaea* L*.,* FJ493289; *Origanum vulgare* L., FJ513100; *Thymus serpyllum* L. DQ667352.

***rps16*** —*Acanthus longifolius* Host, AJ431037; [*Achyrospermum africanum*](http://www.ncbi.nlm.nih.gov/nuccore/FJ854133.1) [Hook.f.](http://www.ipni.org/ipni/idAuthorSearch.do;jsessionid=44E655B352D5E518E7C1EB569476281B?id=4084-1&back_page=/ipni/editSimplePlantNameSearch.do;jsessionid=807A82803C08AAD4D2513DE7839BA78D?find_wholeName=Achyrospermum+africanum&output_format=normal) ex [Baker](http://www.ipni.org/ipni/idAuthorSearch.do;jsessionid=44E655B352D5E518E7C1EB569476281B?id=407-1&back_page=/ipni/editSimplePlantNameSearch.do;jsessionid=807A82803C08AAD4D2513DE7839BA78D?find_wholeName=Achyrospermum+africanum&output_format=normal), FJ853999; *Achyrospermum wallichianum* [Benth. ex Hook.f.](http://www.ipni.org/ipni/idPlantNameSearch.do?id=444198-1&back_page=/ipni/editSimplePlantNameSearch.do?find_wholeName=Achyrospermum+wallichianum&output_format=normal), HQ911594; *Elsholtzia stauntonii* [Benth.](http://www.ipni.org/ipni/idPlantNameSearch.do?id=446697-1&back_page=/ipni/editSimplePlantNameSearch.do?find_wholeName=Elsholtzia+stauntonii&output_format=normal), AJ505406; *Hemigenia pedunculata* [Diels](http://www.ipni.org/ipni/idPlantNameSearch.do?id=447764-1&back_page=/ipni/editSimplePlantNameSearch.do?find_wholeName=Hemigenia+pedunculata&output_format=normal), HQ911570; *Hypenia macrantha* [(Benth.) Harley](http://www.ipni.org/ipni/idPlantNameSearch.do?id=278553-2&back_page=/ipni/editSimplePlantNameSearch.do?find_wholeName=Hypenia+macrantha&output_format=normal), AJ505336; *Lamium galeobdolon* var. montanum, JF780071; *Lamium tomentosum* [Benth. ex Nyman](http://www.ipni.org/ipni/idPlantNameSearch.do?id=448965-1&back_page=/ipni/editSimplePlantNameSearch.do?find_wholeName=Lamium+tomentosum&output_format=normal), JF780105; *Lavandula buchii* [Webb & Berthel.](http://www.ipni.org/ipni/idPlantNameSearch.do;jsessionid=E81D4179F757FC14DB30B5C80D737093?id=449019-1&back_page=/ipni/editSimplePlantNameSearch.do;jsessionid=E81D4179F757FC14DB30B5C80D737093?find_wholeName=Lavandula+buchii&output_format=normal), AJ505346; *Nepeta scordotis* L., HQ911578; *Olea europaea* L., AF225275; *Origanum vulgare* L., JQ716466; *Prostanthera nivea* [Benth.](http://www.ipni.org/ipni/idPlantNameSearch.do?id=156604-3&back_page=/ipni/editSimplePlantNameSearch.do?find_wholeName=Prostanthera+nivea&output_format=normal), AJ505403; *Salvia nilotica* [Murray](http://www.ipni.org/ipni/idPlantNameSearch.do?id=456788-1&back_page=/ipni/editSimplePlantNameSearch.do?find_wholeName=Salvia+nilotica&output_format=normal), HQ911577; *Stachys aculeolata* [Hook.f.](http://www.ipni.org/ipni/idPlantNameSearch.do?id=459260-1&back_page=/ipni/editSimplePlantNameSearch.do?find_wholeName=Stachys+aculeolata&output_format=normal), FJ854084;

*Stachys nephrophylla* [Rech.f.](http://www.ipni.org/ipni/idPlantNameSearch.do?id=459783-1&back_page=/ipni/editSimplePlantNameSearch.do?find_wholeName=Stachys+nephrophylla&output_format=normal), FJ854108; *Stachys spinosa* L., FJ854117.

***trn*L-**trn**F**—[*Achyrospermum africanum*](http://www.ncbi.nlm.nih.gov/nuccore/FJ854133.1) [Hook.f.](http://www.ipni.org/ipni/idAuthorSearch.do;jsessionid=44E655B352D5E518E7C1EB569476281B?id=4084-1&back_page=/ipni/editSimplePlantNameSearch.do;jsessionid=807A82803C08AAD4D2513DE7839BA78D?find_wholeName=Achyrospermum+africanum&output_format=normal) ex [Baker](http://www.ipni.org/ipni/idAuthorSearch.do;jsessionid=44E655B352D5E518E7C1EB569476281B?id=407-1&back_page=/ipni/editSimplePlantNameSearch.do;jsessionid=807A82803C08AAD4D2513DE7839BA78D?find_wholeName=Achyrospermum+africanum&output_format=normal), FJ854133; *Achyrospermum wallichianum* [Benth. ex Hook.f.](http://www.ipni.org/ipni/idPlantNameSearch.do?id=444198-1&back_page=/ipni/editSimplePlantNameSearch.do?find_wholeName=Achyrospermum+wallichianum&output_format=normal), HQ911734; *Elsholtzia stauntonii* [Benth.](http://www.ipni.org/ipni/idPlantNameSearch.do?id=446697-1&back_page=/ipni/editSimplePlantNameSearch.do?find_wholeName=Elsholtzia+stauntonii&output_format=normal), AJ505526

*Hemigenia pedunculata* [Diels](http://www.ipni.org/ipni/idPlantNameSearch.do?id=447764-1&back_page=/ipni/editSimplePlantNameSearch.do?find_wholeName=Hemigenia+pedunculata&output_format=normal), HQ911708, *Hypenia macrantha* [(Benth.) Harley](http://www.ipni.org/ipni/idPlantNameSearch.do?id=278553-2&back_page=/ipni/editSimplePlantNameSearch.do?find_wholeName=Hypenia+macrantha&output_format=normal), AJ505445;

*Lamium galeobdolon* var. montanum, JF779996; *Lamium tomentosum* [Benth. ex Nyman](http://www.ipni.org/ipni/idPlantNameSearch.do?id=448965-1&back_page=/ipni/editSimplePlantNameSearch.do?find_wholeName=Lamium+tomentosum&output_format=normal), JF780032; *Lavandula buchii* [Webb & Berthel.](http://www.ipni.org/ipni/idPlantNameSearch.do;jsessionid=E81D4179F757FC14DB30B5C80D737093?id=449019-1&back_page=/ipni/editSimplePlantNameSearch.do;jsessionid=E81D4179F757FC14DB30B5C80D737093?find_wholeName=Lavandula+buchii&output_format=normal), AJ505460; *Lepechinia calycina* Epl., KF307414;

*Lepechinia urbanii* Epl., KF307433; [*Lepechinia yecorana*](http://www.ncbi.nlm.nih.gov/nuccore/?term=lepechinia) [Henrickson, Fishbein & T.Van Devender](http://www.ipni.org/ipni/idPlantNameSearch.do?id=60456697-2&back_page=/ipni/editSimplePlantNameSearch.do?find_wholeName=Lepechinia+yecorana&output_format=normal), KF307435; *Melissa axillaris* Bakh.f., JQ669051; *Melissa officinalis* L., DQ667477;

*Nepeta scordotis* L., HQ911716; *Olea europaea* L., AF231866; *Origanum vulgare* L., AY570463;

*Physostegia ledinghamii* [(Boivin) P.D.Cantino](http://www.ipni.org/ipni/idPlantNameSearch.do?id=909563-1&back_page=/ipni/editSimplePlantNameSearch.do?find_wholeName=Physostegia+ledinghamii&output_format=normal), EF546873; *Physostegia pulchella* [Lundell](http://www.ipni.org/ipni/idPlantNameSearch.do?id=454195-1&back_page=/ipni/editSimplePlantNameSearch.do?find_wholeName=Physostegia+pulchella&output_format=normal), EF546880; *Prostanthera nivea* [Benth.](http://www.ipni.org/ipni/idPlantNameSearch.do?id=156604-3&back_page=/ipni/editSimplePlantNameSearch.do?find_wholeName=Prostanthera+nivea&output_format=normal), AJ505524; *Salvia nilotica* [Murray](http://www.ipni.org/ipni/idPlantNameSearch.do?id=456788-1&back_page=/ipni/editSimplePlantNameSearch.do?find_wholeName=Salvia+nilotica&output_format=normal), AY570487;

*Stachys aculeolata* [Hook.f.](http://www.ipni.org/ipni/idPlantNameSearch.do?id=459260-1&back_page=/ipni/editSimplePlantNameSearch.do?find_wholeName=Stachys+aculeolata&output_format=normal), FJ854199; *Stachys nephrophylla* [Rech.f.](http://www.ipni.org/ipni/idPlantNameSearch.do?id=459783-1&back_page=/ipni/editSimplePlantNameSearch.do?find_wholeName=Stachys+nephrophylla&output_format=normal), FJ854223; *Stachys spinosa* L., KF529947; *Thymus serpyllum* L., AY570502.
