# Supplementary figures and images for: Insight into Central Asian flora from the Cenozoic Tianshan montane origin and radiation of Lagochilus (Lamiaceae)
Source: PLoS One. 2017 Sep 20;12(9):e0178389. doi: 10.1371/journal.pone.0178389 (PMC5606930; doi:10.1371/journal.pone.0178389)

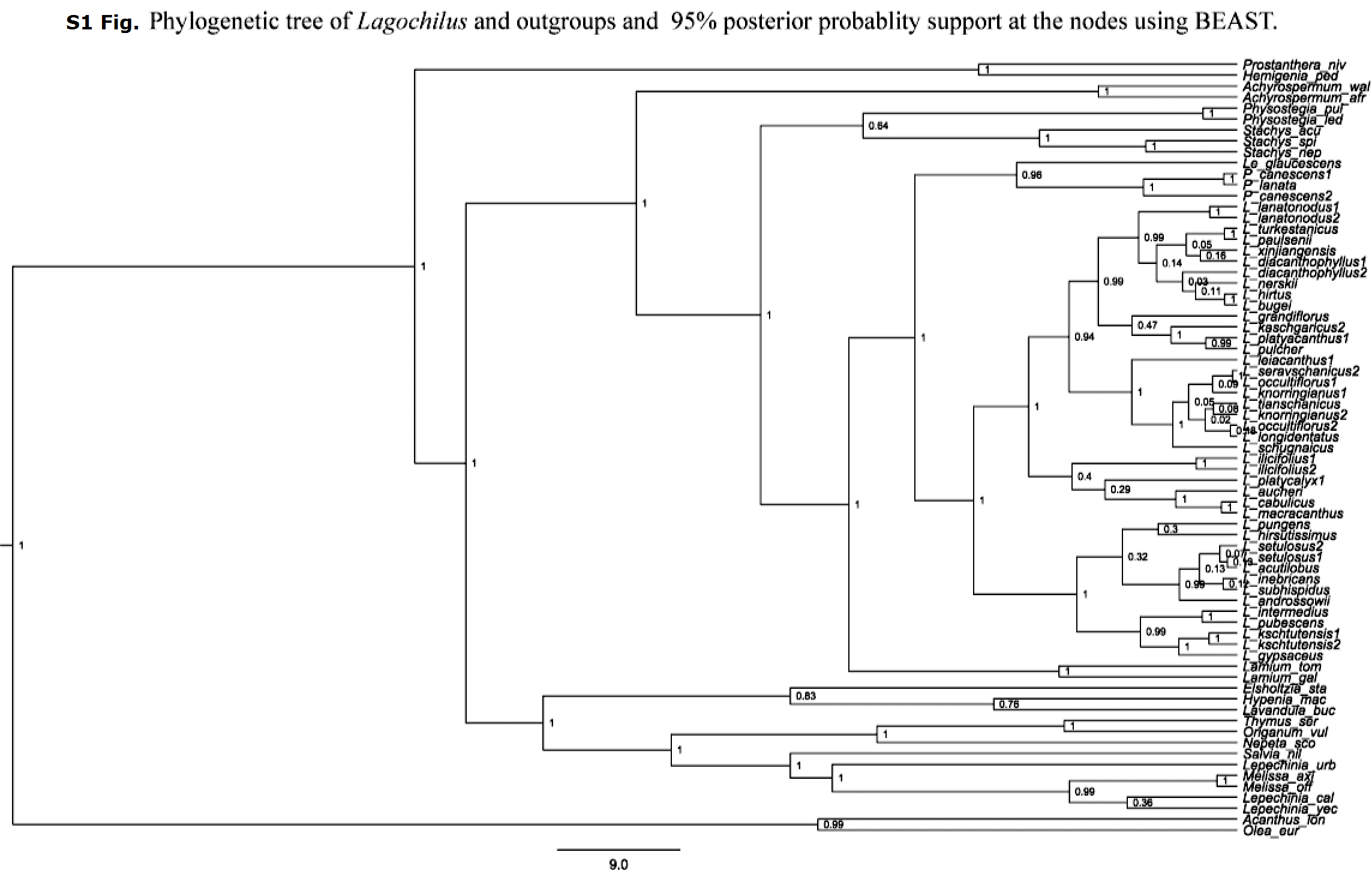

Supplement: S1 Fig — (TIF) [file pone.0178389.s003.tif]
